# PathQC: Determining Molecular and Structural Integrity of Tissues from Histopathological Slides

**DOI:** 10.3390/bioengineering13010005

**Published:** 2025-12-21

**Authors:** Ranjit Kumar Sinha, Anamika Yadav, Sanju Sinha

**Affiliations:** Center for Data Science and Artificial Intelligence, Sanford Burnham Prebys Medical Discovery Institute, San Diego, CA 92037, USA

**Keywords:** digital pathology, quality control, biobanking application

## Abstract

Quantifying tissue, molecular, and structural integrity is essential for biobank development. However, current assessment methods either involve destructive testing that depletes valuable biospecimens or rely on manual evaluations, which are not scalable and lead to interindividual variation. To overcome these challenges, we present PathQC, a deep-learning framework that directly predicts the tissue RNA Integrity Number (RIN) and the extent of autolysis from hematoxylin and eosin (H & E)-stained whole-slide images of normal tissue biopsies. Advancing over prior QC methods focused on imaging quality control, PathQC provides sample-quality control through the direct quantification of molecular integrity (RIN) and structural degradation (autolysis). PathQC first extracts morphological features from the slide using a recently developed digital pathology foundation model (UNI), followed by a supervised model that learns to predict RNA Integrity Number and autolysis scores from these morphological features. PathQC is trained on and applied to the Genotype-Tissue Expression (GTEx) cohort, which comprises 25,306 non-diseased post-mortem samples across 29 tissues from 970 donors, when paired ground-truth RIN and autolysis scores were available. Here, PathQC predicted RIN with an average Pearson correlation of 0.47 and an autolysis score of 0.45, with notably high performance using adrenal gland tissue (R = 0.82) for RIN and colon tissue (R = 0.83) for autolysis. We provide a pan-tissue model for predicting RIN and autolysis scores for new slides from any tissue type (GitHub). Overall, PathQC enables a scalable assessment of tissue molecular and structural integrity from routine H & E images, enhancing biobank quality control and retrospective analyses across 29 tissues and multiple collection sites.

## 1. Introduction

Traditional approaches to tissue-quality assessment rely on molecular metrics, such as the RNA Integrity Number (RIN) [1], or a manual histological evaluation of autolysis [2], both of which require either tissue consumption or extensive expert review. The former is challenging when tissue samples are limited and adds sequencing costs, and the latter introduces inter-individual variations [2,3]. The RNA Integrity Number provides a standardized measure of RNA degradation and directly determines library preparation efficiency and sequencing success, while autolysis reflects the degree of structural breakdown visible under microscopy. Together, they serve as key indicators of biospecimen integrity and usability for molecular profiling.

The digitization of pathology through whole-slide imaging (WSI) technology has transformed how we capture and analyze tissue architecture. These high-resolution digital representations, typically scanned at 20× or 40× magnification, contain rich information about cellular morphology, tissue organization, and potentially, molecular integrity. Current computational approaches to digital pathology primarily address technical *image quality*: scanning artifacts [4], focus quality [5], or staining variations [6]. However, there exists no method that can provide the *sample quality* itself: molecular or structural integrity, despite increasing evidence that image-derived morphological patterns may encode such information. PathQC differs from prior computational pathology efforts by targeting biospecimen quality metrics, rather than disease biomarkers. To our knowledge, this represents the first attempt to predict sample-level molecular and structural integrity directly from routine histopathology slides.

Despite progress in digital pathology and deep learning, no automated framework exists that can assess specimen quality directly from H & E slides, leaving a critical gap in biobank quality control workflows. Current practices either consume tissue for destructive assays or rely on subjective expert scoring, limiting scalability for population-scale biorepositories.

Recent advances, including ours, in computational pathology have revealed that histological images contain far more information than previously recognized [7,8,9,10]. Foundation models trained on millions of pathology images, such as UNI [10], Virchow [11], and Prov-GigaPath [12], have demonstrated remarkable capabilities in extracting meaningful features from tissue morphology. Deep-learning algorithms can now predict mutation status [13], gene expression profiles [14], microsatellite instability [15], telomere length [16], and treatment response directly from H & E-stained slides [17]. These discoveries challenge traditional boundaries between visual and molecular domains, suggesting a deeper connection between what we observe morphologically and what we measure molecularly.

Autolysis, the self-digestion of cells via their own enzymes produces characteristic morphological changes, including nuclear pyknosis, cytoplasmic eosinophilia, and a loss of cellular detail. Pathologists use these patterns to provide an autolysis score [2]. In the same vein, RNA degradation begins immediately after tissue devascularization, triggered by endogenous RNases released from lysosomes. This degradation follows predictable patterns: ribosomal RNA degrades first, followed by mRNA species with varying half-lives [18]. We hypothesize that a systematic analysis of tissue H & E images, paired with molecular RIN and autolysis scores, can help us develop a machine learning model to determine it from H & E images only.

Towards this, we here present PathQC, a computational pathology quality framework to predict RIN number and autolysis scores directly from H & E images. PathQC is trained and applied on the largest corpus of normal tissue data—the Genotype-Tissue Expression (GTEx) cohort [19], with paired H & E and molecular quality metrics. PathQC is designed for assessing non-tumor biospecimen quality in population-scale biobanks. The GTEx project, designed to study tissue-specific gene expression and regulation, provides an ideal foundation for our work with its 25,306 H & E-stained whole-slide images spanning 29 distinct tissue types across approximately 970 individuals.

## 2. Results

### 2.1. Overview of PathQC Pipeline

Multiple current pathology QC methods focus on image quality (scanning artifacts, focus, and overstaining). We present the first method for assessing *specimen quality* itself: molecular and structural integrity from histopathology images, rather than only image-level quality.

**Figure 1:**
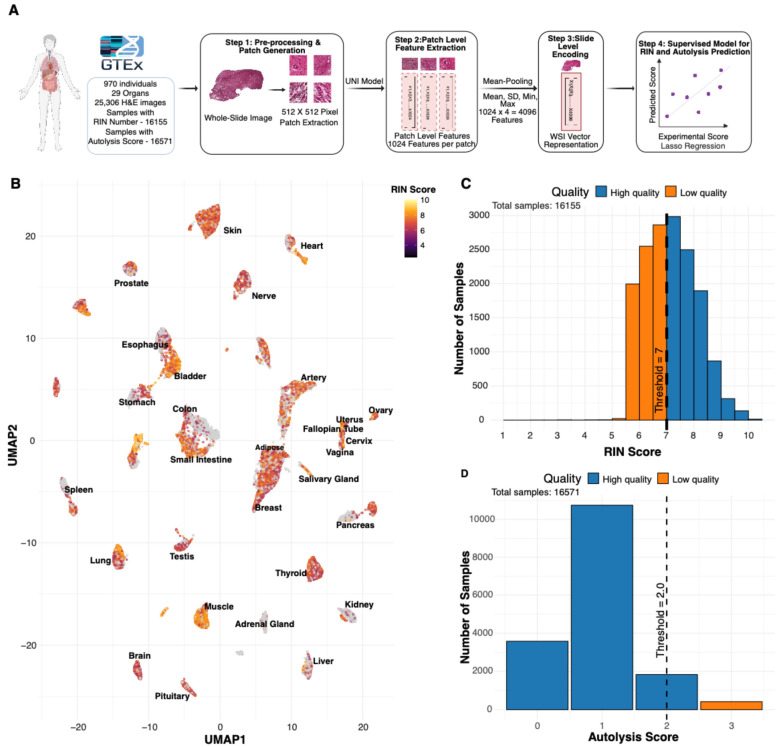
Overview of PathQC framework and tissue-quality landscape. (**A**) PathQC workflow showing preprocessing, feature extraction using UNI encoder, slide-level encoding, and supervised prediction. (**B**) UMAP visualization with RIN score intensity overlay across 29 tissue types showing quality distribution patterns in morphological feature space. (**C**) Distribution of RIN scores across the dataset showed that most samples achieved scores between 6 and 8. (**D**) Distribution of autolysis scores showed most samples had scores of 0–1.

The assessment of biological specimen quality, via RIN number and autolysis scores, requires a destructive sequencing assay and manual pathologist annotation. To overcome this, we present PathQC (Figure 1A) to predict RNA Integrity Number (RIN) and autolysis scores directly from H & E-stained whole-slide images (WSIs). In the GTEx project, histology and RNA-seq aliquots are collected from adjacent pieces of the same tissue block during a single procurement event, ensuring identical donor metadata and pre-analytical handling (warm/cold ischemia time, fixation, and storage). Although these are not the same physical slices, both represent the same tissue environment, allowing PathQC to test whether structural preservation visible on H & E reflects the molecular integrity of the paired sample. The PathQC pipeline comprises four sequential steps (Figure 1A): (1) Slide preprocessing, (2) Morphological feature extraction at the patch level, (3) Generating whole-slide embedding, and (4) Supervised model building to predict RIN and autolysis score based on morphological features. We provide a brief overview of these steps below.

1. **Slide preprocessing:** We begin with the preprocessing of whole-slide images, removing background using Sobel edge detection and applying color normalization using Macenko’s method to mitigate batch effects. Images are segmented into 512 × 512-pixel patches, excluding those with insufficient tissue content (>50% background).
2. **Patch-Level Feature Extraction:** For deep-learning feature extraction, we process these normalized patches through the UNI encoder [10], a vision transformer architecture pre-trained on over 100 million tissue patches across multiple cancer types and normal tissues. This step yields 1024-dimensional feature vectors for each patch, capturing essential visual patterns in each tissue region.
3. **Slide-Level Encoding:** To bridge the resolution gap between patch-level features and slide-level quality metrics, we aggregate patches by taking their average, SD, max, and min, creating a 4096-length vector that creates a comprehensive slide-level feature vector that preserves essential visual information while enabling computational analysis.
4. **Developing a Supervised Model for RIN and Autolysis Scores:** For quality prediction, we developed tissue-specific lasso regression models trained on GTEx cohort described above, comprising whole-slide images with paired RIN and autolysis scores. Their distribution is provided in Figure 1C,D. We compute our model’s performance using five-fold cross-validation; the test set has never seen this data before.

We trained and applied PathQC using cross-validation, computing performance on test data never seen through the model. Our dataset comprised over 30 million patch images from 25,306 whole-slide images across 29 tissue types from 970 donors, all with paired ground-truth RIN and autolysis scores (Figure 1C,D). Our approach is based on the observation that, in a UMAP based on morphological features, high vs. low RIN score samples are clustered separately (Figure 1B and Figure S1). This suggests that morphological features contain information to determine RIN scores, motivating our predictive modeling approach. Training and evaluation were performed within each tissue type, which minimizes but does not eliminate the influence of donor-level and pre-analytical variation (e.g., Hardy scale, post-mortem interval).

### 2.2. Landscape of Tissue Quality Across Different Tissue Types

**Figure 2:**
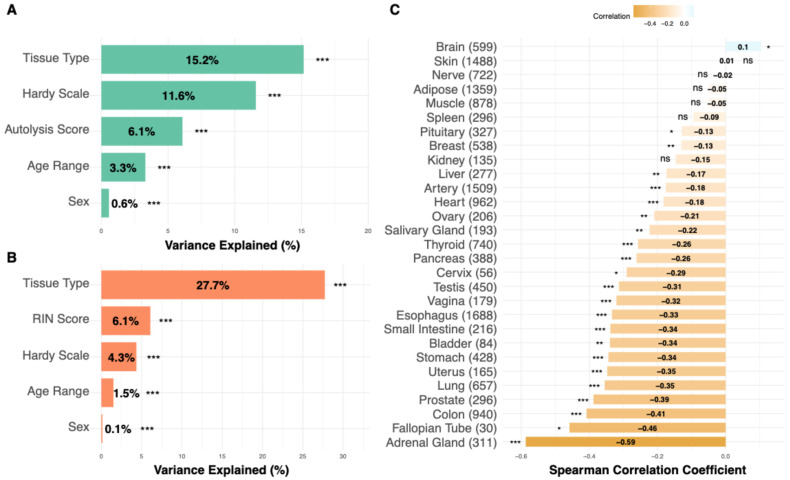
Factors affecting tissue quality in GTEx samples. (**A**) For RIN scores, tissue type explained the largest portion of variance (15.2%), followed by the Hardy scale (circumstances of death) (11.6%), and autolysis score (6.1%). Age and gender had a minimal impact, explaining only 3.3% and 0.6% of variance, respectively. (**B**) For autolysis scores, the tissue type again explained the largest variance proportion (27.7%), followed by the RIN score (6.1%) and the Hardy scale (circumstances of death) (4.3%). (**C**) Cross-tissue correlations between RNA Integrity Number (RIN) and autolysis scores across GTEx samples. Each bar represents the Spearman correlation coefficient for a given tissue, with positive and negative values indicating whether higher RNA integrity is associated with lower or higher autolysis severity, respectively. *Asterisks denote statistical significance* (* *p* < 0.05, ** *p* < 0.01, *** *p* < 0.001; ns *p* ≥ 0.05). In (**C**), bar color intensity encodes the Spearman correlation coefficient.

Before building an H & E-based predictor, we next aim to understand how these RIN and autolysis scores vary in different tissues and their key confounding factors. To this end, we examined their variation across tissue types, demonstrating considerable tissue-specific variation and reflecting intrinsic differences in RNA integrity and degradation susceptibility (Figure S6). We next examined the technical confounders of these two metrics (Figure 2A,B). For RIN scores, the tissue type explained the largest portion of variance (15.2%), followed by the Hardy scale (circumstances of death) (11.6%) and the autolysis score (6.1%) (Figure 2A). Age and gender had a minimal impact, explaining only 3.3% and 0.6% of variance, respectively. For autolysis scores, tissue type explained an even larger variance proportion (27.7%), followed by the RIN score (6.1%) and the Hardy scale (4.3%) (Figure 2B). The analysis demonstrated that age exerted a minimal influence on RIN scores across most tissue types, with tissue-specific variations observed in certain anatomical contexts. These results demonstrate the primary importance of tissue-specific characteristics in determining quality metrics, suggesting that quality assessment approaches should be calibrated to specific anatomical contexts, rather than applied uniformly across tissues. Correlations between RIN and autolysis scores varied significantly across tissues (Figure 2C), with the adrenal gland showing the strongest negative relationship (Spearman r = –0.59), reflecting a monotonic association between the two degradation metrics and indicating that RNA degradation and morphological autolysis are tightly coupled in certain tissue contexts.

Performance trends were consistent across donors differing in the Hardy scale and post-mortem intervals, suggesting that morphology-linked information contributes beyond pre-analytical variation.

### 2.3. H & E Morphology Can Determine RIN and Autolysis Scores in 29 Tissue Types

We next built an H & E morphology-based predictor (fourth step of PathQC), a lasso regression model, using the above-noted sequencing-based RIN number and an expert pathologist’s annotation-based autolysis score in 25,306 GTEx biopsies. Our model was tested in five-fold cross-validation; across five iterations, the model’s performance was evaluated on 20% of the data that had never been seen through the model. PathQC predicted RIN scores across 29 tissues, with an average correlation of 0.47, and autolysis, with an average Pearson correlation of 0.45. These values reflect pooled cross-validated performance within each tissue; independent test-set performance is reported separately in the pan-tissue section below.

**Figure 3:**
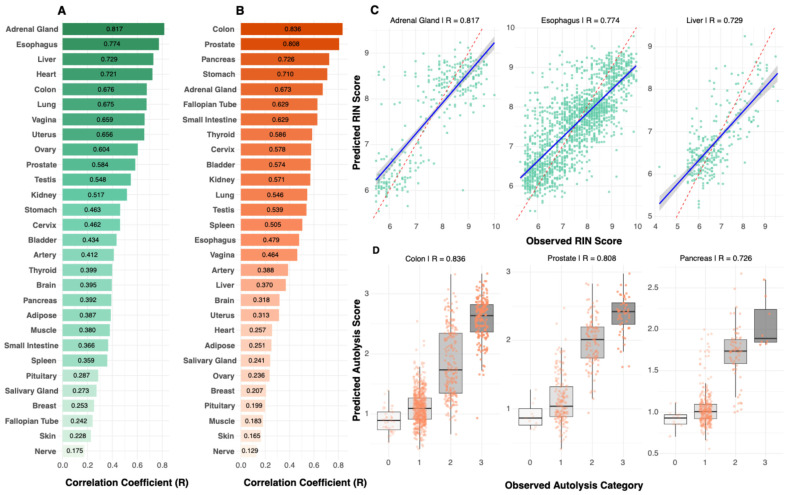
Tissue-specific prediction performance. (**A**) RIN (green) prediction performance varied substantially across tissues, ranging from R = 0.81 (adrenal gland) to R = 0.17 (nerve), demonstrating that tissue biology, rather than sample size, drives predictability. (**B**) Autolysis (orange) prediction performance ranged from R = 0.83 (colon) to R = 0.13 (nerve). Performance patterns revealed tissues with strong prediction for both metrics (colon, adrenal gland), metric-specific performance (esophagus for RIN; prostate for autolysis), and poor prediction for both metrics (nerve, skin). (**C**) Scatter plots for top-performing RIN models showed clear correlations between predicted and observed values. (**D**) Box plots for top-performing autolysis models demonstrated accurate classification into quality categories with minimal overlap between degradation levels. In (**A**,**B**), bar color intensity reflects the magnitude of the correlation coefficient (R).

The prediction performance varies substantially across different tissue architectures, with RIN prediction ranging from 0.81 (adrenal gland), followed by esophagus (R = 0.77, RMSE = 0.75), liver (R = 0.72, RMSE = 0.67), heart (R = 0.72, RMSE = 0.69), to 0.17 (nerve) (Figure 3A and Figure S1). Scatter plots for top-performing tissues (Figure 3C) illustrate the relationship between predicted and actual RIN scores. Similarly, autolysis performance ranged from 0.83 (colon) to 0.13 (nerve) (Figure 3B), and the predicted vs. true autolysis scores are provided for a few tissues in Figure 3D. Comprehensive tissue-specific performance metrics, including correlation coefficients, RMSE values, and sample sizes across all tissues, are provided in Supplementary Table S1. Our model’s cross-validation stability across folds is detailed in Supplementary Note S2 (Figure S7). We also observed a moderate positive association between RIN and autolysis prediction performance (Spearman r = 0.35) (Figure S8A), reflecting a monotonic relationship between the two accuracy metrics and suggesting that these quality measures share some morphological manifestations.

PathQC relies on polysemantic deep-learning features, rather than hand-engineered histologic measurements, so these features are not directly interpretable at this stage. Although vision language models could, in principle, be used to annotate such features, we found them to perform poorly for quantitative quality metrics such as RIN and autolysis and, therefore, did not use them. We did not perform a formal pathologist-driven annotation of degradation patterns in top-performing tissues (e.g., adrenal gland, colon, esophagus, liver), so we cannot rigorously attribute tissue-level performance differences to specific histologic structures in this study. As the field advances, improved tools for interpreting such embeddings will likely emerge, but detailed feature-level interpretability is outside the scope of this work.

In the brain specifically, the available annotations in the independent 30% held-out test set occupy only a narrow band of the autolysis scale. Most samples are labeled with intermediate degradation scores (1–2), whereas well-preserved sections (score 0) and maximally degraded sections (score 3) are absent. This compressed mid-range label distribution restricts the variance in ground-truth autolysis and dampens the apparent correlation between predicted and observed values for the brain, even though the sample size is adequate (Figure S13).

To evaluate whether performance was driven primarily by the best-preserved specimens, we performed a preservation–percentile analysis based on RIN using pan-tissue PathQC predictions on the same independent 30% test set. For each tissue, we sorted samples by observed RIN and progressively removed the highest-quality samples, recomputing the Pearson correlation between predicted and observed RIN at successive preservation percentiles. In the esophagus, which has the largest number of RIN-evaluable test samples, correlations remained stable across a wide range of percentiles and only declined when the analysis was restricted to the most degraded samples (Figure S13). Table S2 extends this analysis to all 29 tissues and shows that, in most organs, PathQC retains meaningful predictive signal even when higher-quality samples are excluded, indicating that performance is not driven solely by a small subset of optimally preserved slides.

### 2.4. Pan-Tissue Model Jointly Predicts Both Integrity Measures

**Figure 4:**
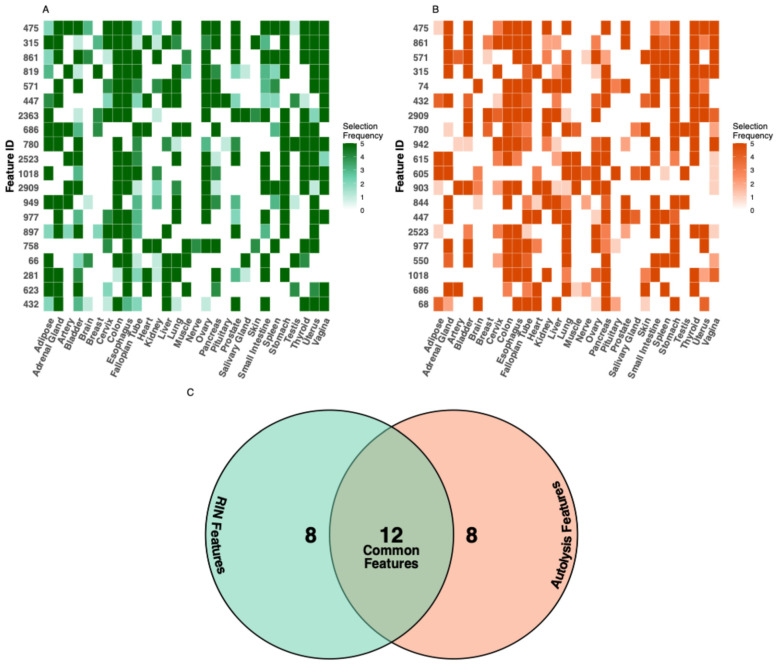
Feature importance analysis across tissue types. The feature importance heatmap illustrates the tissue-specificity of predictive features for (**A**) RIN and (**B**) autolysis prediction. (**C**) Comparing features important for both quality metrics, we identified 12 common features that showed high importance for both RIN and autolysis prediction across multiple tissues. These shared features suggest common morphological manifestations of degradation that affect both RNA integrity and general tissue preservation. Additionally, eight features showed high specificity for RIN prediction, and another eight features showed specificity for autolysis prediction, indicating that these quality aspects also manifest through distinct morphological changes.

Probing important features shared across tissue types, we identified the 20 most important features across all tissues for each integrity task (methods, Figure 4A,B). Hierarchical clustering revealed that morphologically similar tissues utilize similar feature sets. Further, 12 out of 20 top features were shared between RIN and autolysis models (Figure 4C). This prompted us to develop a pan-tissue model that can take any tissue type, H & E, and jointly predict both RIN and autolysis scores. This can be useful for an individual to use PathQC without relying on choosing tissue-specific models.

**Figure 5:**
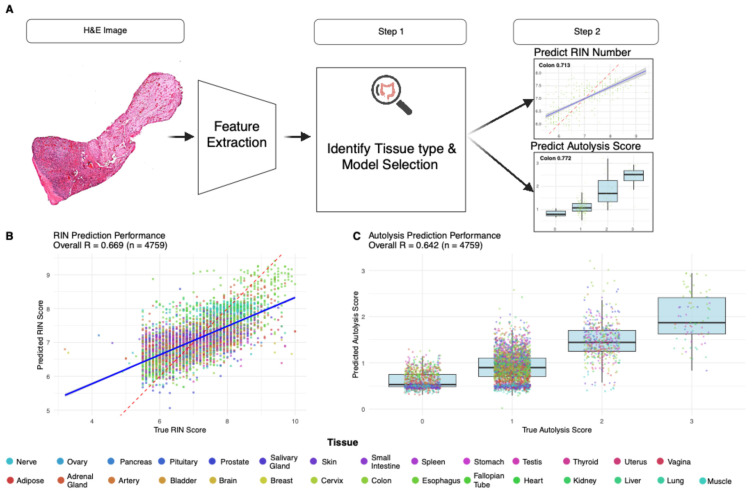
Pan-tissue model performance and workflow. (**A**) Pan-tissue PathQC framework implementing a two-stage approach: tissue type classification, followed by tissue-specific quality prediction, achieving 99.2% tissue classification accuracy with samples routed to appropriate tissue-specific models based on predicted tissue type. (**B**) Pan-tissue RIN prediction achieved R = 0.67 (RMSE = 0.70) across 99.6% of test samples, with several tissues demonstrating consistent performance across both training and test sets. (**C**) Pan-tissue autolysis prediction observed 70.1% prediction accuracy and R = 0.64 correlation, with tissues like the colon and the prostate showing robust morphological patterns for quality prediction across quality metrics. Legend colors indicate tissue type.

To develop a pan-tissue model, we implemented a two-stage approach through which we first predicted the tissue type and then accordingly selected a tissue-specific model for prediction (Figure 5A). To this end, for our first step of tissue-type prediction, we developed a multinomial Lasso regression, where the top 500 most informative features are used (F-score). Our model predicted the right tissue type with 99.2% overall accuracy in 5-fold stratified CV (macro-averaged AUC > 99.9%). Pairwise AUC analysis demonstrated exceptional tissue discrimination, with values ranging from 0.97 to 1.00 across all tissue pairs, indicating near-perfect separability based on morphology.

To clearly distinguish evaluation settings, cross-validated results refer only to within-training-set performance under 5-fold stratification, whereas final performance metrics (for both RIN and autolysis) are computed on the independent 30% held-out test set (n = 4759) using Pearson correlation, which is never used during cross-validation. All test-set results reported below correspond exclusively to this held-out evaluation.

Following tissue classification, samples were routed to appropriate tissue-specific models. The pan-tissue RIN prediction achieved R = 0.67 (RMSE = 0.70) across 99.6% of test samples (Figure 5B). For autolysis prediction, we observed 70.1% prediction accuracy and R = 0.64 correlation (Figure 5C). Several tissues demonstrated consistent performance across both training and test sets, including the colon and the prostate, suggesting robust morphological patterns for quality prediction across quality metrics (Figure 5B,C). Comprehensive training and test performance metrics across all tissues within the pan-tissue framework, including correlation coefficients, sample sizes, and RMSE values for both RIN and autolysis predictions, are provided in Table S1.

Because the pan-tissue framework spans 29 distinct organs collected across multiple sites, it inherently serves as a proxy for cross-dataset and multi-institutional generalization. The consistent performance across tissues suggests that PathQC generalizes beyond any single dataset or acquisition condition.

## 3. Discussion

Building on the recent advancements in the digital pathology foundation model to extract morphological features, we provide the first computational framework to predict RIN and autolysis scores and tissue integrity measures from H & E images. For biobanking workflows, PathQC adoption will enable the rapid quality screening of large collections, preserve limited material for research, rather than quality control, and facilitate a retrospective assessment of historical collections in which molecular quality data may be unavailable. In particular, a pan-tissue single model is applicable for most tissues and thus will help in adoption. Given that only 4% of total H & E samples in the U.S. are currently scanned, and this will only increase, the adoption of digital pathology models and their applications will increase. PathQC serves as a basis for automated quality control using scanned images.

PathQC differs fundamentally from prior AI-based quality control frameworks that focus on imaging quality, such as stain variation, focus, or scanner quality (Haghighat et al., 2022 [4]). Here, the objective is to estimate specimen quality, capturing degradation within the tissue itself, rather than image quality on the slide, and thereby extend quality assessment from image fidelity to biospecimen integrity. This distinction introduces a new dimension of quality control, linking histologic structure directly to specimen integrity and complementing existing image-based QC pipelines.

Despite its promising results, PathQC faces three key limitations: (1) The prediction performance varies substantially across tissue types, with some tissues showing excellent prediction while others remain challenging. (2) PathQC predicts bulk-tissue-quality metrics, rather than spatial quality variation within samples, where tissue degradation often occurs heterogeneously. (3) The interpretability of deep-learning features remains challenging despite our feature importance analysis (Figure 4A,B and Figure S5).

Model performance is not uniform across tissues. In the tissue-specific models (Figure 3A,B), nerve shows the lowest performance (RIN R = 0.17; autolysis R = 0.13), with skin also in the bottom tier for both metrics. The pan-tissue training results show the same pattern (RIN: skin R = 0.43, nerve R = 0.45; autolysis: skin R = 0.23, nerve R = 0.22). In contrast, adrenal gland, esophagus, liver, heart, colon, prostate, and gynecologic tissues reach R ≥ 0.70 for at least one endpoint, indicating that PathQC is most reliable in these tissues and should be interpreted cautiously for nerve and skin. Consistent with this, our preservation-percentile analysis on the held-out test set (Figure S13, Table S2) shows that PathQC retains predictive signal even when the best-preserved specimens are removed, supporting its utility for triaging lower-quality samples in realistic biobank settings.

A key strength of PathQC is that it achieved high performance without additional color normalization beyond Macenko’s preprocessing, without information on Hardy scale or time before preservation. Although histologic and RNA aliquots in GTEx are adjacent, rather than identical sections, both share identical pre-analytical histories, supporting a meaningful morphology–molecular link. Future extensions incorporating same-block adjacent sections or spatial transcriptomic data could enable pixel-level correspondence between morphology and molecular integrity, further resolving whether the observed associations reflect intrinsic tissue preservation or donor-level variation.

Although GTEx tissues are largely non-diseased, biobank specimens often contain necrosis, inflammation, or other pathological alterations that can mimic or obscure degradation-related morphology. Necrosis may resemble severe autolysis, and inflammatory infiltrates may disrupt features relevant to RIN prediction, meaning PathQC could partially conflate pathology with degradation. Incorporating training data with controlled pathological variability will be important for improving generalization to clinical biobanks.

In its current form, PathQC is intended as a decision-support tool for tissue-quality assessment, helping biobanks identify and exclude low-quality specimens before sequencing. The most promising direction for PathQC beyond addressing these limitations with either technical development or additional data modalities is the development of models that can conceptually take a step beyond predicting tissue degradation toward restoring or correcting it. Analogous to image-enhancing models in computer vision, enhancing damaged photos, one can learn from the large corpus of high-quality images and degrade them artificially to create paired labels. This conceptual breakthrough can help biobanks overcome tissue damage during preparation or scanning in H & E images at least.

Because the framework spans 29 tissues across multiple institutions (pan-tissue analysis), it is well positioned for generalization to diverse biobank settings. Broader validation studies across different sample preparation protocols and imaging platforms will be essential for establishing PathQC’s generalizability and clinical utility, paving the way for wider adoption in biobanking and clinical workflows.

Although the average Pearson correlation of approximately 0.47 for RIN prediction may appear modest, it is operationally meaningful in biobanking workflows. Even moderate correlation reliably distinguishes low-quality samples from well-preserved ones, enabling biobanks to triage degraded tissues prior to sequencing and reduce downstream assay failures. As such, PathQC provides actionable sample-quality insight even in tissues where fine-grained prediction is challenging.

## 4. Methods

### 4.1. Acquiring GTEx Dataset and Quality Metrics

Genotype-Tissue Expression (GTEx) v10 was downloaded from https://gtexportal.org/home/API (accessed on 20 January 2025). Each digitized at a high resolution (20× magnification, ~0.5 microns per pixel), these H & E-stained slides represent one of the largest collections of paired histological–molecular data available. For paired data, autolysis scores were assigned by expert pathologists using a four-point ordinal scale: 0 (none), 1 (slight), 2 (moderate), and 3 (severe), based on digitally scanned whole-slide images created using (Aperio) [20]. RIN scores were determined using the Agilent 2100 Bioanalyzer system (Agilent Technology, Santa Clara, CA, USA) to quantify RNA integrity. Slides with missing RIN or autolysis values were excluded from their respective prediction tasks, and slides that failed preprocessing due to insufficient tissue content (fewer than two valid patches after background filtering) were removed prior to analysis. GTEx sample collection and pathology review procedures are described in Carithers et al. (2015) [21].

In the analytic dataset used for PathQC, each observation corresponds to a single whole-slide image (WSI). A given donor–tissue pair contributes between one and five WSIs, and this sampling pattern is tissue-specific. All 421 brain donor–tissue pairs are represented by exactly two slides, whereas esophagus donor–tissue pairs most commonly contribute two or three slides (approximately 16% with two slides, 81% with three slides, and 2% with four or five slides). In most other tissues, donor–tissue pairs are represented by a single slide, with only a small minority contributing more than one slide.

Pairing H & E and RNA-Seq based RIN Score: Each donor–tissue block in GTEx is subdivided during a single procurement event, with one aliquot allocated to histology (H & E staining) and parallel aliquots reserved for RNA extraction. According to the GTEx Tissue Processing Center, H & E and RNA aliquots are adjacent, rather than identical, sections; they share identical pre-analytical handling, potentially allowing morphological features to reflect molecular integrity. These procedures are described in detail in the GTEx Consortium study [22].

### 4.2. PathQC Pipeline

PathQC comprises the following four sequential steps, for which the first three generating slide-level encoders were originally designed in Yadav, Alvarez et al. (2025) [16]. However, we provide their brief description here for completeness.

1. Image preprocessing: We first prepared the WSIs by segmenting them into 512 × 512-pixel patches after background removal using Sobel edge detection. RGB color normalization was performed using Macenko’s method to reduce staining variability across slides digitized at multiple GTEx collection sites under different scanners. Across all GTEx slides, this preprocessing yielded over 30 million valid tissue patches, with an average of approximately 1200 patches per slide.
2. Patch-level feature extraction: A digital foundation model, UNI, a pre-trained transformer-based encoder, was used to extract 1024-dimensional feature vectors capturing morphology patterns in each patch.
3. Generating slide-level embedding: Given that our quality metrics were at the slide level, we generated slide-level embedding to train a predictive model for them. Each whole-slide image was divided into **P** non-overlapping patches, and for each patch, ***P***, we extracted a d-dimensional feature vector, ***z_sp_*** ∈ ${\mathbb{R}}^{d}$ from the pretrained UNI foundation model. To obtain a single representation per slide, we employed a combined aggregation strategy that captures both central tendencies and variability in patch features. The aggregated slide-level feature vector was defined as follows:
  **x**_s_ = [mean_p_(**z**_sp_), std_p_(**z**_sp_), min_p_(**z**_sp_), max_p_(**z**_sp_)]
  where each operator is applied element-wise over feature channels. This produces a four-dimensional embedding (4096 features for d = 1024) that captures both global morphology (mean) and within-slide heterogeneity (standard deviation, minimum, maximum). Collecting these slide-level embeddings across all *N* slides yields a feature matrix, ***X*** ∈ **ℝ*^N x 4d^***, where each row corresponds to one whole-slide image (WSI). Slides derived from the same donor–tissue pair, therefore, appear as separate rows and are treated as independent observations. All cross-validation and test-set evaluations are performed at the slide level, rather than the donor level. This matrix was standardized and used as input to a Lasso regression model:
  **ŷ** = **X**β, β* = arg min_β_ [1/(2N) ||**y** − **X**β||_2_^2^ + λ||β||_1_]
  where λ was selected via five-fold cross-validation and **α** = 1. This linear multi-instance learning formulation treats each slide as a bag of patches, summarizing region-level morphological variability through simple yet interpretable statistics while preserving the nonlinear structure learned during UNI pretraining.
4. Supervised training: We next aimed to predict molecular quality metrics from the above-created slide-level embedding. To this end, we developed tissue-specific lasso regression models for both RIN and autolysis scores. Lasso regression was chosen for its ability to perform feature selection while building the predictive model, effectively handling the high-dimensional feature space while producing sparse, interpretable models. All features were z-scored, and zero-variance features were removed. The model was implemented using the (glmnet) package in R with an L1 regularization parameter (**α** = 1). The optimal penalty term (**λ**) was determined through 5-fold cross-validation based on the minimum mean-squared error (**λ_min_**). Model performance was quantified using Pearson correlation computed on pooled cross-validated predictions from held-out folds. For each tissue, the final model was retrained on the full training split using the optimal **λ** and evaluated once on the held-out 30% test set, which had not been seen during any cross-validation step. Within each fold, the top 5% of features most correlated with the target were selected before model fitting. Model performance was assessed using the correlation coefficient (R) between predicted and observed values on the independent test set.

### 4.3. Feature Importance and Sharing Analysis

To analyze feature-sharing patterns across tissues, we extracted feature selection frequencies from all tissue-specific models (Figure 4A,B). For each target (RIN and autolysis), we aggregated feature selection counts across all tissues and identified the 20 most frequently selected features globally.

Feature usage patterns were visualized using heatmaps showing selection frequency by tissue type. To identify relationships between tissue types based on feature usage, we performed hierarchical clustering on both features (rows) and tissues (columns) using Euclidean distance and complete linkage. Feature overlap between RIN and autolysis models was quantified by comparing the top 20 feature sets from each prediction task.

### 4.4. Model Stability and Cross-Validation Analysis

To evaluate model consistency and reliability across folds, we conducted stability analysis across cross-validation folds by calculating the coefficient of variation (CV) of correlation coefficients for each tissue-specific model:

**CV** = **σ**/**μ**

where σ is the standard deviation, and μ is the mean of correlation coefficients across folds. Models with CV values below 0.2 were classified as stable, while those exceeding 0.5 were considered unstable. Because our analysis involved a limited number of tissues and did not rely on formal hypothesis testing across features or tissues, we did not apply multiple-testing correction and report unadjusted performance metrics.

### 4.5. Pan-Tissue Model Development

To enable quality prediction across diverse tissue types, we developed a hierarchical pan-tissue framework consisting of tissue classification followed by tissue-specific quality prediction. Dataset preparation: We combined samples from all 29 tissue types with both RIN and autolysis scores (n = 16,117), applying a 70:30 train–test split stratified by tissue type and RIN quartiles. Only samples with complete RIN and autolysis labels were retained for the pan-tissue model. This stratification ensures balanced representation of tissues and quality score ranges in both training and test partitions.

1. Tissue classification. ANOVA F-score selection identified the top 500 discriminative features across tissue types. We trained a multinomial lasso regression model using stratified 5-fold cross-validation with L1 regularization (**α** = 1).
2. Quality prediction. Samples were routed to tissue-specific models based on predicted tissue type and a confidence threshold (0.6). Separate lasso regression models for RIN and autolysis were trained for each tissue using all 4096 features with stratified cross-validation by RIN quartiles and autolysis categories, respectively. Performance metrics for the pan-tissue framework were computed on pooled predictions from the independent held-out 30% test set using Pearson correlation.

Performance evaluation: Tissue classification was assessed using accuracy, macro F1-score, and macro-AUC. Quality prediction employed correlation coefficients for RIN and classification accuracy, along with correlation for autolysis. Coverage was calculated as the proportion exceeding the confidence threshold. All metrics for the pan-tissue framework are reported at the slide level.

## Data Availability

The data used in this study were obtained from the Genotype-Tissue Expression (GTEx) Project (accession: phs000424.v10.p2, Project titled #39103). The H & E histological images are publicly available and were accessed through the GTEx Portal v10 release (https://gtexportal.org/home/ (accessed on 20 January 2025). Autolysis Score and RIN score were also obtained from GTEx Portal (https://www.gtexportal.org/home/downloads/adult-gtex/metadata/ (accessed on 20 January 2025). Individuals’ ages were extracted after appropriate institutional approval and data access agreement (General Research Use: phs000424.v10.p2.c1). All processed data for figure generation is provided in Supplementary Tables. The underlying code for this study is available on GitHub and can be accessed via https://github.com/Cranjit9/PathQC (accessed on 20 January 2025).

## Supplementary Materials

The following supporting information can be downloaded at https://www.mdpi.com/article/10.3390/bioengineering13010005/s1, Table S1. Complete Performance. Table S2: Preservation vs. Model Performance. Figure S1. UMAP visualization of top-performing tissue quality prediction models. Figure S2. Sample size effects on model performance across tissue types. Figure S3. Feature consistency and cross-tissue analysis. Figure S4. Model residual analysis and prediction accuracy validation. Figure S5. Feature importance analysis and morphological pattern characterization. Figure S6. Quality score distributions across major tissue types. Figure S7. Cross-validation stability and model robustness evaluation. Figure S8. Comparative model analysis and cross-metric relationships. Figure S9. Model performance comparison across top-performing tissues. Figure S10. Comprehensive analysis of tissue morphological patterns and dataset characteristics. Figure S11. Model performance vs In-fold consistency. Figure S12. Pan-tissue model performance validation across test data. Figure S13. Brain prediction, behavior, and preservation-percentile analysis.
